# Improving Balance and Gait in Older People with Parkinson’s Disease: A Randomized Controlled Trial of Technology-Assisted Rehabilitation Interventions

**DOI:** 10.3390/bioengineering13050487

**Published:** 2026-04-23

**Authors:** Elvira Maranesi, Roberta Bevilacqua, Elisa Casoni, Ilaria Barboni, Federico Barbarossa, Arianna Margaritini, Chiara Polverigiani, Arianna Sgolastra, Emanuela Bertino, Renato Baldoni, Marco Benadduci, Giulio Amabili, Pietro Scendoni, Giuseppe Pelliccioni, Valentina Di Donna, Giovanni R. Riccardi

**Affiliations:** 1 Scientific Direction, IRCCS INRCA, 60124 Ancona, Italy; e.maranesi@inrca.it (E.M.); f.barbarossa@inrca.it (F.B.); a.margaritini2@inrca.it (A.M.); a.sgolastra@inrca.it (A.S.); m.benadduci@inrca.it (M.B.); g.amabili@inrca.it (G.A.); 2 Clinical Unit of Physical Rehabilitation, IRCCS INRCA, 60124 Ancona, Italy; e.casoni@inrca.it (E.C.); i.barboni@inrca.it (I.B.); c.polverigiani@inrca.it (C.P.); e.bertino@inrca.it (E.B.); r.baldoni@inrca.it (R.B.); g.riccardi@inrca.it (G.R.R.); 3 Clinical Unit of Physical Rehabilitation, IRCCS INRCA, 63900 Fermo, Italy; p.scendoni@inrca.it (P.S.); v.didonna@inrca.it (V.D.D.); 4 Neurology Unit, IRCCS INRCA, 60124 Ancona, Italy; g.pelliccioni@inrca.it

**Keywords:** older people, Parkinson’s disease, technology, balance, gait rehabilitation

## Abstract

(1) Background: Parkinson’s disease (PD) is a neurodegenerative disorder characterized by gait and postural impairments. Recently, physical activity has emerged as a key strategy in PD management. This study aimed to evaluate the effectiveness of an innovative technology-assisted rehabilitation program in improving gait and reducing fall risk in older adults with PD. (2) Methods: Fifty-eight patients were randomly assigned to three groups: conventional rehabilitation (CG), or conventional therapy combined with technology-assisted rehabilitation using Tymo (TG) or Walker View (WG). The intervention consisted of 10 sessions over 5 weeks. Assessments were conducted at baseline (T0), post-intervention (T1), and at 6-month follow-up (FW). Outcomes included gait and balance performance, fear of falling, quality of life, activities of daily living, and physical function. (3) Results: The CG showed no significant improvements, with a decline in Barthel Index from T1 to FW. The WG demonstrated significant improvement in POMA Gait scores, while the TG improved both POMA Total and Balance scores at T1. Post-treatment, TG and WG outperformed CG in POMA outcomes; however, these differences were lost at follow-up. (4) Conclusions: Technology-assisted rehabilitation can improve gait and balance in older adults with PD, although sustained or repeated interventions may be necessary to maintain long-term benefits.

## 1. Introduction

Parkinson’s disease (PD) is a prevalent neurological disorder, with an estimated global prevalence exceeding 6 million cases in 2015, making it the neurological disease with the most rapidly increasing prevalence [1]. Its occurrence is particularly notable in the aging population, affecting approximately 1% of adults over the age of 60 [2]. This disease is characterized by a highly heterogeneous spectrum of manifestations and ages of onset, resulting in unique symptom profiles in individual patients [3]. Among the primary motor impairments associated with PD are bradykinesia (slowness in movement), postural instability, rigidity, and resting tremor. One common manifestation is difficulty in lifting the feet while walking, leading to small, shuffling steps, that significantly increases the risk of falls and diminishes the overall quality of life [4].

Another significant challenge faced by individuals with PD is maintaining postural stability, as reduced stability contributes to increased disability, fall risk, and decreased quality of life [5,6]. Both balance and postural stability play crucial roles in the performance of daily activities and are fundamental aspects of overall mobility among people with PD [7,8].

In recent years, physical activity and exercise have emerged as crucial interventional strategies for PD management [9,10,11,12]. Exercise, as an integral component of physical therapy, focuses on improving physical fitness, transfer skills, manual dexterity, balance, and gait to enhance motor function and movement independence in PD patients [9,13]. The volume, type, and intensity of exercise are key determinants of its beneficial outcomes. Evidence suggests that high-intensity exercise may be particularly advantageous in the early stages of PD compared to low-intensity training [14]. Moreover, individualized training programs, combined with motivation and feedback, are likely to further enhance the benefits of exercise in patients with PD.

Various training programs, including gait and balance training lasting for at least several weeks (e.g., 4 weeks for gait and 8 weeks for balance), as well as regular resistance, exercise, or dance interventions, have demonstrated long-lasting benefits on strength, balance, and walking capacities [15]. A comprehensive review from 2016 concluded that exercise consistently improves general physical and cognitive capacities in people with PD, leading to enhanced functional abilities. Specifically, improvements in gait efficiency, velocity, cadence, balance, posture, and fall risk were among the most robustly reported outcomes [16].

In recent years, there has been an increasing interest in technological innovations to augment traditional rehabilitation paradigms and address the multifaceted challenges posed by PD [17,18,19]. The integration of technology into rehabilitation holds promise for providing personalized, engaging, and effective interventions tailored to the unique needs of patients. From wearable sensors and virtual reality systems to robotics and mobile applications, a wide range of technological tools have emerged as potential adjuncts to conventional therapy, offering novel avenues for enhancing motor performance, promoting neuroplasticity, and facilitating long-term functional gains. Numerous studies have underscored the potential benefits of technology-assisted rehabilitation in PD. For instance, a randomized controlled trial by Mirelman et al. (2016) [20] demonstrated that a virtual reality treadmill training program led to significant improvements in gait and balance parameters compared to conventional treadmill training in individuals with PD. Similarly, a systematic review by Tao et al. (2023) [21] highlighted the efficacy of robot-assisted therapy in enhancing upper limb function and reducing motor impairment in PD patients.

This randomized controlled trial aims to evaluate an innovative technology-assisted rehabilitation therapy for older patients with Parkinson’s disease, designed to improve gait and reduce fall risk. Specifically, the study investigates the effectiveness of two technology-based rehabilitation interventions, the Tymo system and Walker View (WV), in improving gait and balance in older patients with PD.

This study advances current knowledge on technology-assisted rehabilitation in Parkinson’s disease by directly comparing two distinct technological approaches—one targeting gait kinematics (Walker View) and one targeting postural control (Tymo)—within the same randomized controlled design. Unlike previous studies that typically evaluate single devices in isolation, this work clarifies how different technological focuses translate into specific functional outcomes and includes a 6-month follow-up to assess durability of effects.

## 2. Materials and Methods

This research presents preliminary data from the study titled “Innovative Models in the Rehabilitation of Elderly Patients with Parkinson’s disease Through Technological Innovation.” The trial was officially registered in the ClinicalTrials.gov database under the identifier NCT04087031 on 10 September 2019. The study was conducted in accordance with the principles outlined in the Declaration of Helsinki and received approval from the Ethics Committee of Istituto di Ricerca a Carattere Scientifico—Istituto Nazionale di Ricovero e Cura per Anziani—IRCCS INRCA (approval number CE19017, issued on 25 July 2019). The study adheres to the CONSORT guidelines.

This trial is a single-blinded, randomized controlled study with blinded outcome assessors; however, both participants and physiotherapists were unblinded. Informed consent was obtained from all enrolled individuals before participation. Data collection started in January 2020 and remains ongoing.

### 2.1. Subjects

A total of 58 individuals with PD were recruited through the outpatient department of the Clinical Unit of Physical Rehabilitation at IRCCS INRCA, at both the Ancona and Fermo sites. The participants were randomly assigned to one of three groups: a control group (CG) undergoing conventional rehabilitation; a Tymo group (TG) receiving conventional therapy augmented with Tymo system-assisted rehabilitation; or a Walker View group (WG) receiving conventional therapy augmented with Walker View system-assisted rehabilitation, using a computer-generated randomization sequence with a 1:1:1 allocation ratio. The randomization list was created prior to enrolment using a computer-based random number generator, and group allocation was implemented sequentially after each participant provided informed consent, ensuring unbiased assignment and preventing selection bias. Evaluations were conducted at baseline, post-intervention, and six months post-treatment.

Inclusion criteria were: age over 65 years; ability to provide informed consent; Hoehn and Yahr (H&Y) stage 1–3 [22]; Functional Ambulation Category (FAC) score ≥ 2 [23]; Rankin Scale (RS) score [24] ≤ 3; stable dopaminergic medication regimen for ≥1 month; negative result on the Geriatric Depression Scale (GDS-5) [25]; and a Mini Mental State Examination (MMSE) score ≥ 24 [26]. Eligibility was assessed during recruitment; eligible participants then provided informed consent and underwent baseline evaluation. Participants were excluded if they did not meet the inclusion criteria, were concurrently enrolled in other studies, or failed to provide written informed consent. Further exclusion criteria were: a Clinical Dementia Rating (CDR) score ≥ 3; a history of syncopal episodes, epilepsy, or vertigo not adequately controlled by pharmacological treatment; severe autonomic dysfunction; severe behavioral disorders not adequately managed with medication; concomitant neurological diseases; severe systemic diseases with a life expectancy of less than 1 year; and inability to complete follow-up.

Baseline assessments included the Clinical Dementia Rating Scale (CDR) [27], technology acceptance measured by the Psychosocial Impact of Assistive Devices Scale (PIADS) [28], functional status assessed by the Barthel Index (BI) [29], and gait/balance evaluated using Tinetti’s Performance Oriented Mobility Assessment (POMA) [30]. Additionally, gait speed during free walking was measured using a G-Walking sensor, quality of life was assessed through the SF-12 Health Survey [31], and fear of falling was measured with the Falls Efficacy Scale—International (FES-I) [32].

### 2.2. Intervention

The rehabilitation program consisted of 10 sessions delivered twice weekly over 5 weeks. Participants in the CG underwent conventional therapy sessions lasting 50 min each. Individuals in the TG and WG received 30 min of standard rehabilitation plus 20 min of treatment with the respective technological system.

All participants performed conventional rehabilitation exercises, targeting six specific functional domains: transfers (bed mobility, sit-to-stand); posture (postural alignment and control); reaching and grasping (upper extremity function); balance (static and dynamic balance control); gait (walking speed, stride length, freezing of gait, effective toe clearance); physical capacity (joint mobility, muscle power, endurance); and compensatory Strategy Approaches (visual, auditory and tactile cues or breaking down complex movements into component parts).

The TG intervention utilized the Tymo^®^ system, a wireless platform delivering non-immersive virtual reality exergames. These exercises were customized to match each participant’s functional abilities, aiming to improve balance and postural control.

The WG intervention utilized the Walker View system, a treadmill equipped with a sensorized belt containing eight load cells and a 3D camera to measure step length, speed and symmetry, as well as trunk, hips and knee range of motion. Patients walked at a comfortable speed while the physiotherapist adjusted parameters such as step length, load distribution, and step height, providing the patient with visual and auditory feedback to enable real-time gait correction.

Each system targets distinct motor components relevant to gait and postural control recovery. Specifically, WV targets gait kinematics and symmetry, providing real-time visual feedback on spatiotemporal parameters such as step length, stance and swing phase durations, and step height. Through repetitive gait training, it promotes step symmetry, both spatial (such as equalizing left-right step lengths) and temporal (such as balancing stance/swing times), and facilitates symmetrical load distribution, encouraging more physiological weight acceptance on both limbs during ambulation. It also analyzes gait phases and deviations to normalize step trajectories, including foot clearance and propulsion. In contrast, the Tymo system targets postural control and balance strategies, addressing both static and dynamic balance to reduce postural sway and improve weight-shifting abilities. The intervention emphasizes mediolateral and anteroposterior load transfers, training patients to modulate movements effectively and enhance anticipatory and reactive balance control. These components are crucial for tasks such as initiation of gait, single-limb support, and transitioning between postures, which are often impaired in individuals with neurological disorders.

Table 1 summarizes the key similarities and differences among the interventions.

### 2.3. Statistical Analysis

Continuous variables were reported as either mean ± standard deviation (SD) or median and interquartile range (IQR), based on their distribution, assessed using the Shapiro–Wilk test. Between-group comparisons of continuous variables were performed using an unpaired Student’s *t*-test or Mann–Whitney U test, according to their distribution. Categorical variables were expressed as frequencies (n) and percentages, and analyzed using Pearson’s Chi-square test or Fisher’s exact test, as appropriate.

Follow-up data were analyzed using multivariate statistical techniques to evaluate intervention effectiveness and compare changes over time in outcome measures among intervention groups and controls. For repeated measures, data distribution determined the test selection: a repeated-measures ANOVA was used for normally distributed data, and the Friedman test for non-normally distributed data. Statistical significance was set at *p* < 0.05. Within-group effect sizes (Cohen’s d) were calculated to estimate the magnitude of changes between timepoints (T0–T1 and T1–FW). The pooled standard deviation method was used, with effect sizes interpreted using Cohen’s conventional thresholds: 0.2 (small), 0.5 (medium), ≥0.8 (large). Additionally, a repeated-measures multivariate ANOVA (MANOVA; 3 × 3 design: Group × Time) was performed to assess group-time interactions. All statistical analyses were performed using SPSS (version 28) and Matlab (version R2020b) software.

## 3. Results

**Figure 1:**
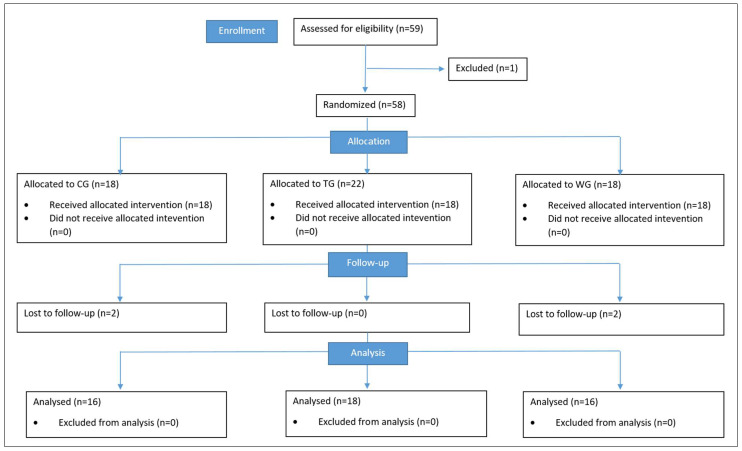
The CONSORT flowchart.

The CONSORT (Consolidated Standards of Reporting Trials) flowchart is shown in Figure 1.

Demographic, clinical and functional data of the sample (18 subjects for CG, 18 subjects for WG and 22 subjects for TG) are reported in Table 2. At baseline, there were no differences found between the two groups in terms of inclusion criteria values and demographic characteristics.

Table 3 shows pre- and post-intervention scores (at the end of treatment and at 6-month follow-up) and intra group differences between the beginning and end of treatment, between the end of treatment and follow-up, and between the start of the training and the follow-up of each group on the scales used to assess the impact of the rehabilitation treatments.

**Figure 2:**
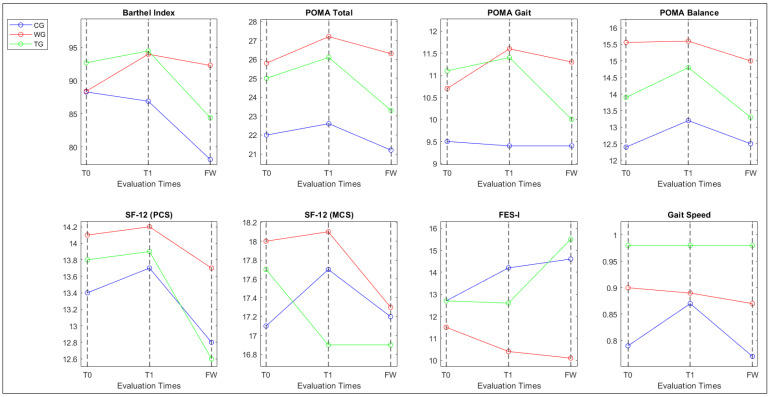
Evolution of key outcome measures across T0, T1, and FW for each group. Data for the control group (CG) are shown in blue, the Walker View group (WG) in red, and the Tymo group (TG) in green.

Figure 2 shows the evolution of key outcome measures across T0, T1, and FW for each group.

Table 3 shows that, in the CG, there are no significant improvements in the BI, POMA Total, POMA Gait, or POMA Balance scores from baseline to post-intervention (T0–T1). However, a significant decline in BI is observed from post-intervention to follow-up (T1–FW), indicating a deterioration in daily living activities over time. Similarly, no significant changes are found in the SF-12 physical and mental component scores, FES-I, or gait speed. In the WG, a significant improvement is detected in the POMA Gait score from baseline to post-intervention (T0–T1), suggesting enhanced gait performance. No significant changes are reported in the BI, POMA Total, POMA Balance, SF-12 scores, FES-I, or gait speed across the intervention and follow-up periods. The TG shows significant improvements in the POMA Total and POMA Balance scores from baseline to post-intervention (T0–T1), indicating better balance control. Additionally, significant declines in POMA Total, POMA Gait, and POMA Balance scores are observed from post-intervention to follow-up (T1–FW), suggesting a partial loss of the gains achieved during the intervention. The BI also shows a significant decline at follow-up, while no significant changes are found in the SF-12 scores, FES-I, or gait speed. Moreover, no significant differences are observed between baseline and follow-up (T0–FW) in any of the three groups for the analyzed outcome measures, except for the Barthel Index.

Effect sizes were calculated for outcome measures that showed a significant or clinically relevant change across the three time points (T0, T1, FW), in line with the goal of evaluating the intervention’s impact over time. The CG did not exhibit meaningful improvements across time points. Effect sizes from T0 to T1 were negligible for most outcomes, including POMA Total (d = 0.11), POMA Gait (d = −0.04), and Barthel Index (d = −0.09). At follow-up (T1–FW), a moderate negative effect was found in the Barthel Index (d = −0.51), suggesting functional decline over time. The WG showed moderate effect sizes in functional mobility from baseline to post-intervention (T0–T1), particularly in the POMA Gait (d = 0.66) and POMA Total (d = 0.50). A small effect was also observed in the Barthel Index (d = 0.44). However, these gains were only partially maintained at follow-up, with smaller effect sizes observed between T1 and FW (e.g., POMA Gait d = 0.40; BI d = 0.26). Minimal or negligible changes were detected in POMA Balance (d = 0.03) and other measures. The TG showed small-to-moderate effect sizes from baseline to post-intervention (T0–T1) in measures such as POMA Balance (d = 0.47) and POMA Total (d = 0.38). At follow-up, effect sizes indicated moderate declines in POMA Gait (d = −0.50) and BI (d = −0.54), suggesting partial attenuation of treatment effects over time.

Table 4 reports group-by-time interactions.

Table 4 assesses the group × time interactions as well as the independent main effects of group and time. To evaluate changes in outcomes over time and the interaction between groups and time, a repeated-measures ANOVA (3 × 3 design: Group × Time) was conducted. The analysis revealed a significant main effect of Time (F(2, DF) = 6.57, *p* = 0.0022; Greenhouse-Geisser corrected *p* = 0.0054), indicating that performance on the outcome measures changed over time across all groups. However, no significant main effect of Group was found (F(2, DF) = 1.44, *p* = 0.249), and the Group × Time interaction was not significant (F(4, DF) = 0.84, *p* = 0.503), suggesting that the patterns of change over time did not differ statistically between the groups.

## 4. Discussion

The primary objective of this study was to evaluate the effectiveness of two technology-assisted rehabilitation devices, the Tymo^®^ system and Walker View, in improving gait and balance in older patients with PD. While our results demonstrate statistically significant within-group improvements in specific gait and balance metrics post-intervention (T1), a key aspect in interpreting these findings is determining their clinical meaningfulness, beyond mere statistical significance. For the POMA scale, the literature indicates a minimal clinically important difference (MCID) of approximately 1–3 points in populations with balance impairments and high fall risk. In our study, the WG showed a mean increase of 0.9 points in POMA Gait from T0 to T1, at the lower boundary of this range, while the TG achieved 0.9 points in POMA Balance and 1.1 points in POMA Total, modest but potentially clinically meaningful gains in postural control, consistent with the targeted mechanisms of each device [33,34]. These changes, though not sufficient to reclassify fall risk, reflect functional enhancements aligned with therapeutic goals. Notably, no clinically meaningful improvements were observed in gait speed (10 mWT) across groups, consistent with prior studies on treadmill-based training in PD, where maintenance of baseline speed represents a positive outcome amid disease progression. Similarly, worsening FES-I scores at follow-up (particularly in TG) suggest no sustained reduction in fear of falling, underscoring perceptual challenges. However, these short-term gains were not sustained at six-month follow-up in the TG and CG, with only the WG maintaining POMA Gait benefits. This highlights the challenges of long-term retention in progressive PD, emphasizing the need for booster interventions. Our findings align with previous research suggesting that technology-assisted rehabilitation can enhance motor performance in PD patients [15,35,36]. For instance, Mirelman et al. (2016) [20] demonstrated that virtual reality treadmill training improved gait and balance more than conventional treadmill training. Similarly, Tao et al. (2023) [21] reported that robot-assisted therapy effectively enhanced upper limb function and reduced motor impairment in PD patients. Our study extends these findings by showing that specific systems, such as Tymo and Walker View, can also significantly improve gait and balance in older PD patients.

The short-term improvements observed in the groups using the two devices can be attributed to the specific nature of the interventions. Walker View provides real-time visual and auditory feedback on gait symmetry and spatio-temporal parameters, enabling patients to correct errors and optimize performance during training. Tymo, on the other hand, targets dynamic balance through interactive games that train anticipatory and reactive postural adjustments. These targeted mechanisms of action, in contrast to traditional physiotherapy, likely contributed to the functional improvements observed after training. Indeed, the significant improvements observed in the Tymo and Walker View groups suggest that incorporating technology-assisted devices into rehabilitation programs for older PD patients can lead to marked enhancements in functional mobility. However, unlike some studies that reported long-term benefits, our results indicate a regression of these improvements at the follow-up. This underscores the need for continuous or repeated interventions to sustain the benefits of technology-assisted rehabilitation. Although some groups showed clinically significant improvements after the intervention (particularly in balance and gait measures) and subsequent partial declines at follow-up, the lack of a significant group × time interaction suggests that these time trends did not statistically differ between groups. Nevertheless, the observed within-group improvements (particularly in the TG and WG at T1), along with short-term between-group differences favoring TG and WG over CG at T1, support the clinical relevance of the interventions despite the absence of a statistically significant interaction in the repeated-measures analysis.

The fact that these improvements were not maintained at follow-up, as evidenced by the lack of significant differences between baseline and follow-up assessments, highlights the potential need for ongoing or booster sessions to sustain the benefits. This finding is crucially relevant, suggesting that a one-time intervention may not be sufficient for lasting improvements. Clinicians may need to consider integrating periodic re-evaluations and additional therapy sessions to reinforce initial gains. Moreover, incorporating home-based exercises by mobile applications or wearable devices could provide real-time feedback and personalized adjustments, fostering adherence and promoting long-term benefits. A hybrid model combining technology-assisted rehabilitation with supervised home exercises may also address logistical and resource constraints while enhancing patient autonomy.

The decline in functional gains at follow-up raises important questions about the mechanisms underlying the loss of benefits. Several factors may contribute to this phenomenon. PD is a progressive neurodegenerative disorder, and the natural course of the disease may overshadow the benefits obtained through short-term interventions. Additionally, discontinuation of the structured rehabilitation program may lead to reduced physical activity levels and subsequent decline in motor performance. Ongoing engagement in therapeutic activities may therefore be essential to maintain the improvements. Patients may also adapt to the initial gains and without continued challenges or progression in the rehabilitation program, benefits may plateau or regress. Although these improvements are clinically modest according to the reported MCIDs, they support the use of these devices in targeted rehabilitation programs.

Despite promising clinical results, the implementation of technology-assisted rehabilitation must also be evaluated from a cost-effectiveness perspective. Devices such as Tymo and Walker View require considerable upfront investment, dedicated space and specific staff training. Although these systems offer advantages such as real-time feedback and personalized task-oriented training, economic viability remains a critical issue requiring targeted studies. Previous economic evaluations suggest that technology-assisted therapy may be cost-effective in specific populations, such as post-stroke patients [37,38,39], but comparable studies in PD populations are still limited. Further research is needed to determine whether the observed short-term functional benefits justify the costs of large-scale application in public health settings.

From a feasibility perspective, technology-assisted rehabilitation may pose practical challenges in public health settings. Limited access to technology and the need for specialized personnel may hinder integration into routine care. In addition, healthcare systems may struggle to prioritize expensive interventions in the absence of long-term outcomes data. To mitigate these barriers, scalable solutions such as mobile rehabilitation units, shared equipment models or partnerships with other rehabilitation centers could be explored.

These factors suggest that a more dynamic and continuous approach to rehabilitation might be necessary. To address the issue of maintaining functional gains, future research should focus on long-term interventions, investigating the effects of prolonged and continuous technology-assisted rehabilitation programs, potentially incorporating periodic booster sessions. Exploring the integration of technology-assisted devices with other therapeutic modalities, such as pharmacotherapy, cognitive training, or conventional physical therapy, may further enhance and sustain benefits. It would also be valuable to examine the long-term effects of combining technology-assisted rehabilitation with general exercise programs such as Nordic walking, Tai Chi, or dance [40,41,42], or even simple home-based treadmill walking. If results are encouraging, this could have major implications for health policy regarding chronic disease management and patient self-care.

Furthermore, it should be noted that even within technology-assisted rehabilitation, task-oriented exercise has been shown to improve specific performance components [43], suggesting that combining Tymo and Walker View in rehabilitation program may enhance effects on both dynamic and static balance, as reflected in the POMA results at the end of the intervention.

According to our data, individuals who underwent technological rehabilitation showed a worse perception of fear of falling at follow up compared with T0. This finding highlights the need to maintain rehabilitation intervention over time, as inactivity may negatively affect perceived stability and confidence in walking.

Regarding walking speed, our results do not show an improvement in any of the three groups analyzed. This is consistent with previous studies comparing the use of RAGT with treadmill training in PD patients using the 10 mWT scale, which also found no significant improvement in gait speed over time [44,45].

Both balance and treadmill training do not appear to influence walking speed; however, these findings should not be interpreted negatively. The maintenance of walking speed following all types of training is, in itself, a positive outcome. Findings from a previous study [46] show that FES-I scores are strongly associated with other balance measures. In our data, we observed the worst results at 6-month follow-up in the Tymo group compared with the WV and control groups. These results may reflect a subjective perception of imbalance at follow-up among individuals trained exclusively with Tymo, suggesting that balance-focused re-education may induce greater changes in balance-related daily living skills. The fear of falling may be perceived as worsening after a period without specific balance training.

It would also be beneficial to leverage technological advancements, such as machine learning and adaptive algorithms, to create more responsive and personalized rehabilitation programs capable of adjusting in real-time to the patient’s performance and progression.

This study presents several limitations that should be acknowledged. First, the sample size was relatively small (58 participants divided into three groups), which may limit the statistical power to detect subtle between-group differences. As noted in the manuscript, “no significant differences are observed between baseline and follow-up (T0–FW) in any of the three groups for the analyzed outcome measures,” suggesting that the study may have been underpowered to capture long-term effects. Additionally, the follow-up period of six months, although clinically meaningful, may not be sufficient to fully understand the durability of technology-assisted rehabilitation benefits, especially considering the progressive nature of Parkinson’s disease. A further limitation concerns the single-blinded design of the study. Although outcome assessors were blinded, participants and physiotherapists were necessarily aware of group allocation, which may have introduced some degree of performance or expectation bias. This aspect is particularly relevant in rehabilitation research, where motivation and therapist–patient interaction can influence short-term functional performance. Nonetheless, the use of standardized assessment procedures and blinded evaluators helped ensure that the measurement of gait and balance outcomes remained objective and consistent across groups.

Future research should address these limitations through larger, multi-center randomized controlled trials to improve generalizability and statistical power. Given the lack of sustained improvements with technology-assisted interventions at follow-up, studies should evaluate booster sessions or continuous, lower-intensity maintenance programs to preserve functional gains. Personalized protocols adapting intensity and task specificity to individual motor profiles—leveraging real-time feedback from systems like Walker View^®^ and Tymo^®^—would also be valuable. Moreover, building on these findings, future work should incorporate advanced biomechanical analyses, including spatiotemporal and kinematic gait metrics beyond simple speed (as measured here via 10 mWT). Integrating wearable sensors or instrumented motion-capture systems could yield high-resolution data to complement clinical scales like POMA, providing deeper insights into motor control mechanisms in PD and enhancing the interpretation of treatment effects.

## 5. Conclusions

This randomized controlled trial shows that technology-assisted rehabilitation can provide short-term improvements in specific gait and balance parameters in older adults with Parkinson’s disease. The Walker View system was associated with improvements in gait performance, while the Tymo system primarily enhanced balance-related outcomes at the end of treatment. However, these benefits were not consistently maintained at the 6-month follow-up, and the overall group-by-time interaction was not significant, indicating that the trajectories of change did not differ statistically between groups. These findings suggest that technology-assisted interventions may complement conventional rehabilitation by targeting specific motor components, but sustained or repeated training may be necessary to preserve gains over time. Further research with larger samples and extended intervention protocols is needed to clarify the long-term clinical relevance of these technologies in Parkinson’s disease rehabilitation.

## Figures and Tables

**Table 1:** Similarities and differences among the interventions.

Aspects	CG	WG	TG
**Session Duration**	50 min	30 min conventional + 20 min WV therapy	30 min conventional + 20 min Tymo therapy
**Number of sessions**	10 sessions (2×/week for 5 weeks)	10 sessions (2×/week for 5 weeks)	10 sessions (2×/week for 5 weeks)
**Technological device**	N/A	Walker View (sensorized treadmill with 3D camera)	Tymo (non-immersive VR balance platform)
**Technological focus**	N/A	Gait kinematics, symmetry, load distribution	Postural control, static and dynamic balance
**Type of feedback**	N/A	Real-time visual and auditory feedback on gait parameters	Real-time visual feedback through exergames
**Targeted motor** **components**	General mobility and coordination	Gait symmetry (step length, stance/swing phases, foot clearance, propulsion)	Balance strategies (weight shifting, postural sway, anticipatory/reactive balance control)
**Training specificity**	Non-specific, general functional training	Task-specific gait training with repetition and parameter-focused feedback	Task-specific balance training using mediolateral and anteroposterior load transfers
**Therapist role**	Full session guided by therapist	Therapist monitors and adjusts gait parameters on WV	Therapist customizes exergames and provides support during balance tasks

CG = control group; TG = Tymo group; WG = Walker View Group; WV = Walker View; VR = Virtual reality; N/A = not applicable.

**Table 2:** Baseline demographic and clinical profile.

	CG	WG	TG	*p*
	*n* = 18	*n* = 18	*n* = 22	
Gender, *n* (%)				0.758
Female	8 (44.5%)	8 (44.5%)	12 (45.5%)	
Male	10 (55.5%)	10 (55.5%)	10 (54.5%)	
Age, mean ± SD ^†^	75 ± 5.9	73.4 ± 6.8	72.8 ± 5.8	0.486
Marital status, *n* (%)				0.940
Married	15 (83.3%)	14 (77.7%)	18 (81.8%)	
Widowed	2 (11.1%)	2 (11.1%)	4 (18.2%)	
Separated/Divorced	1 (5.6)	1 (5.6%)	0 (0%)	
Single	0 (0%)	1 (5.6%)	0 (0%)	
Educational level, *n* (%)				0.613
Primary education	7 (38.8%)	5 (27.8%)	11 (50%)	
Secondary education	8 (44.5%)	8 (44.5%)	6 (27.3%)	
University or more	3 (16.7%)	5 (27.8%)	5 (22.7%)	
Hoehn and Yahr score, mean ± SD	1.9 ± 0.9	1.7 ± 0.7	1.8 ± 0.7	0.097
Rankin Scale score, mean ± SD	1.9 ± 1.1	1.4 ± 1.0	1.2 ± 0.6	0.112
GDS, mean ± SD	1.6 ± 1.3	1.2 ± 1.0	1.4 ± 1.5	0.058
FAC, mean ± SD	4.6 ± 0.9	4.3 ± 0.8	4.6 ± 0.5	0.116
MMSE, mean ± SD ^†^	26.5 ± 2.5	27.3 ± 1.8	27.6 ± 1.9	0.209

*n* = number; CG = control group; TG = Tymo group; WG = Walker View Group; SD = standard deviation; GDS = Geriatric Depression Scale; FAC = Functional Ambulation Category; MMSE = Mini Mental State Examination; ^†^ = parametric test.

**Table 3:** Mean ± standard deviation of the mean of pre-intervention, post-intervention and follow-up scores within the follow up on the BI, POMA (total, gait and balance), SF-12 (total, physical and mental component score), FES-I and gait speed. Pre/post and post/follow-up comparisons are reported for each score (*p* < 0.05).

**PANEL** **A—CG**	**T0**	**T1**	**FW**	***p*** **-Value** **T0–T1**	***p*** **-Value** **T1–FW**	***p*** **-Value** **T0–FW**
BI	88.3 ± 16.0	86.9 ± 16.9	78.1 ± 23.4	0.472	0.039 *	0.0297 *
POMA						
POMA Total	22.0 ± 4.9	22.6 ± 6.2	21.2 ± 7.8	0.419	0.269	0.789
POMA Gait	9.5 ± 2.1	9.4 ± 3.1	9.4 ± 3.2	0.740	0.337	0.396
POMA Balance	12.4 ± 3.0	13.2 ± 3.1	12.5 ± 4.1	0.069	0.288	0.539
SF-12						
PCS-12	13.4 ± 2.0 ^†^	13.7 ± 2.2 ^†^	12.8 ± 2.0	0.497	0.783	0.459
MCS-12	17.1 ± 1.9	17.7 ± 1.7	17.2 ± 1.4	0.460	0.210	0.564
FES-I	12.7 ± 5.9	14.2 ± 6.9	14.6 ± 5.9	0.141	1.000	0.164
Gait Speed [m/s]	0.79 ± 0.31 ^†^	0.87 ± 0.36 ^†^	0.77 ± 0.26	0.091	0.085	0.423
**PANEL B—WG**	**T0**	**T1**	**FW**	***p*** **-value** **T0–T1**	***p*** **-value** **T1–FW**	***p*** **-value** **T0–FW**
BI	88.4 ± 15.8	94.0 ± 8.7	92.3 ± 14.5	0.140	0.485	0.111
POMA						
POMA Total	25.8 ± 3.7	27.2 ± 1.4	26.3 ± 2.6	0.077	0.100	0.149
POMA Gait	10.7 ± 1.8	11.6 ± 0.7	11.3 ± 1.1	0.043 *	0.334	0.052
POMA Balance	15.56 ± 2.1	15.6 ± 0.9	15.0 ± 1.6	0.155	0.065	0.411
SF-12						
PCS-12	14.1 ± 1.5 ^†^	14.2 ± 1.6 ^†^	13.7 ± 1.7	0.879	0.240	0.199
MCS-12	18.0 ± 1.3	18.1 ± 1.6	17.3 ± 2.3	0.515	0.354	0.094
FES-I	11.5 ± 4.0	10.4 ± 3.7	10.1 ± 3.9	0.254	0.582	0.100
Gait Speed [m/s]	0.90 ± 0.21 ^†^	0.89 ± 0.21 ^†^	0.87 ± 0.22	0.786	0.392	0.953
**PANEL C—TG**	**T0**	**T1**	**FW**	***p*** **-value** **T0–T1**	***p*** **-value** **T1–FW**	***p*** **-value** **T0–FW**
BI	92.7 ± 10.4	94.5 ± 13.3	84.4 ± 19.2	0.401	0.031 *	0.371
POMA						
POMA Total	25.0 ± 3.2	26.1 ± 2.5	23.3 ± 5.8	0.023 *	0.013 *	0.552
POMA Gait	11.1 ± 1.5	11.4 ± 1.0	10.0 ± 2.7	0.300	0.009 *	0.120
POMA Balance	13.9 ± 2.1	14.8 ± 1.7	13.3 ± 3.3	0.005 *	0.027 *	0.689
SF-12						
PCS-12	13.8 ± 1.6 ^†^	13.9 ± 1.2 ^†^	12.6 ± 3.2	0.797	0.111	0.150
MCS-12	17.7 ± 1.6	16.9 ± 1.8	16.9 ± 1.4	0.107	0.451	0.158
FES-I	12.7 ± 5.4	12.6 ± 5.4	15.5 ± 6.3	0.923	0.253	0.359
Gait Speed [m/s]	0.98 ± 0.30 ^†^	0.98 ± 0.36 ^†^	0.98 ± 0.31	0.996	0.865	0.865

CG = control group; TG = Tymo group; WG = Walker View group; T0 = baseline; T1 = end of the treatment; FW = follow-up, 6 months after the end of the treatment; BI = Barthel Index, POMA Gait = Tinetti’s Performance Oriented Mobility Assessment-Gait part; POMA Balance = Tinetti’s Performance Oriented Mobility Assessment-Balance part, SF-12 = SF-12 health survey; SF-12-Tot = SF-12 health survey total score; PCS-12 = SF-12 physical component score; MCS-12 = SF-12 mental component score; FES-I = Falls Efficacy Scale—International; * *p*-values from matched-pair Student’s *t* test; ^†^ = parametric test.

**Table 4:** Group-time interactions.

	MeanSq	F	*p* Value
**Group**	181.77	1.44	0.249
**Group × Time**	15.23	0.84	0.503

## Data Availability

The data associated with the paper are not publicly available but are available from the corresponding author on reasonable request.

## Abbreviations

The following abbreviations are used in this manuscript:

SF-12	12-item Short Form Survey
BI	Barthel Index
CDR	Clinical Dementia Rating Scale
CG	control group
FAC	Functional Ambulation Category
FES-I	Falls Efficacy Scale—International
FW	follow-up
GDS	Geriatric Depression Scale
H&Y	Hoen and Yahr
IQR	interquartile range
MCID	minimal clinically important difference
MMSE	Mini Mental State Examination
n	number
PD	Parkinson’s disease
PIADS	Psychosocial Impact of Assistive Devices Scale
POMA	Tinetti’s Performance Oriented Mobility Assessment
RS	Rankin Scale
SD	standard deviation
T0	baseline
T1	end of the treatment
TG	Tymo group
WG	Walker View group

## References

[1] Dorsey E.R., Sherer T., Okun M.S., Bloem B.R. (2018). The emerging evidence of the Parkinson pandemic. J. Park. Dis..

[2] Tolosa E., Garrido A., Scholz S.W., Poewe W. (2021). Challenges in the diagnosis of Parkinson’s disease. Lancet Neurol..

[3] Kalia L.V., Lang A.E. (2015). Parkinson’s disease. Lancet.

[4] Kim S.M., Kim D.H., Yang Y., Ha S.W., Han J.H. (2018). Gait Patterns in Parkinson’s Disease with or without Cognitive Impairment. Dement. Neurocogn. Disord..

[5] Dibble L.E., Addison O., Papa E. (2009). The effects of exercise on balance in persons with Parkinson’s disease: A systematic review across the disability spectrum. J. Neurol. Phys. Ther..

[6] Li S., Chen S., Yu X., Quan M., Deng R. (2026). Scoping review of AI-driven wearable technologies for rehabilitation and functional assessment in Parkinson’s disease: A protocol. BMJ Open.

[7] Kim S.D., Allen N.E., Canning C.G., Fung V.S.C. (2013). Postural instability in patients with Parkinson’s disease. CNS Drugs.

[8] Sarter M., Albin R.L., Kucinski A., Lustig C. (2014). Where attention falls: Increased risk of falls from the converging impact of cortical cholinergic and midbrain dopamine loss on striatal function. Exp. Neurol..

[9] Bouça-Machado R., Rosário A., Caldeira D., Caldas A.C., Guerreiro D., Venturelli M., Tinazzi M., Schena F., Ferreira J.J. (2020). Physical activity, exercise, and physiotherapy in Parkinson’s disease: Defining the concepts. Mov. Disord. Clin. Pract..

[10] Feng Y.S., Yang S.D., Tan Z.X., Wang M.M., Xing Y., Dong F., Zhang F. (2020). The benefits and mechanisms of exercise training for Parkinson’s disease. Life Sci..

[11] Goodwin V.A., Richards S.H., Taylor R.S., Taylor A.H., Campbell J.L. (2008). The effectiveness of exercise interventions for people with Parkinson’s disease: A systematic review and meta-analysis. Mov. Disord. Off. J. Mov. Disord. Soc..

[12] Fernandes J.V.A., Figueiredo V.L.F.A., Oliveira A.B., Reis I.A., Henrique G.L.D., da Silva E.A. (2025). Virtual reality in Parkinson’s disease: A systematic review and meta-analysis. Dement. Neuropsychol..

[13] Bartolo M., Castelli A., Calabrese M., Buttacchio G., Zucchella C., Tamburin S., Fontana A., Copetti M., Fasano A., Intiso D. (2024). A wearable system for visual cueing gait rehabilitation in Parkinson’s disease: A randomized non-inferiority trial. Eur. J. Phys. Rehabil. Med..

[14] Schenkman M., Moore C.G., Kohrt W.M., Hall D.A., Delitto A., Comella C.L., Josbeno D.A., Christiansen C.L., Berman B.D., Kluger B.M. (2018). Effect of highintensity treadmill exercise on motor symptoms in patients with de novo Parkinson disease: A phase 2 randomized clinical trial. JAMA Neurol..

[15] Mak M.K., Wong-Yu I.S., Shen X., Chung C.L. (2017). Long-term effects of exercise and physical therapy in people with Parkinson disease. Nat. Rev. Neurol..

[16] Lauz’e M., Daneault J.F., Duval C. (2016). The Effects of physical activity in parkinson’s disease: A review. J. Park. Dis..

[17] Das J., Morris R., Barry G., Celik Y., Godfrey A., McDonald C., Walker R., Vitorio R., Stuart S. (2023). Technological solution for the assessment and rehabilitation of visuo-cognition in Parkinson’s disease. Expert. Rev. Med. Devices.

[18] Maranesi E., Casoni E., Baldoni R., Barboni I., Rinaldi N., Tramontana B., Amabili G., Benadduci M., Barbarossa F., Luzi R. (2022). The Effect of Non-Immersive Virtual Reality Exergames versus Traditional Physiotherapy in Parkinson’s Disease Older Patients: Preliminary Results from a Randomized-Controlled Trial. Int. J. Environ. Res. Public Health.

[19] Zhou J., Salvendy G., Boot W.R., Charness N., Czaja S., Gao Q., Holzinger A., Ntoa S., Rau P.-L.P., Rogers W.A. (2025). Grand Challenges of Smart Technology for Older Adults. Int. J. Hum.–Comput. Interact..

[20] Mirelman A., Rochester L., Maidan I., Del Din S., Alcock L., Nieuwhof F., Rikkert M.O., Bloem B.R., Pelosin E., Avanzino L. (2016). Addition of a non-immersive virtual reality component to treadmill training to reduce fall risk in older adults (V-TIME): A randomised controlled trial. Lancet.

[21] Tao Y., Luo J., Tian J., Peng S., Wang H., Cao J., Wen Z., Zhang X. (2023). The role of robot-assisted training on rehabilitation outcomes in Parkinson’s disease: A systematic review and meta-analysis. Disabil. Rehabil..

[22] Hoehn M., Yahr M. (1967). Parkinsonism: Onset, progression and mortality. Neurology.

[23] Holden M.K., Gill K.M., Magliozzi M.R. (1986). Gait assesment for neurologically imparired. Standards for outcome assessment. Phys. Ther..

[24] van Swieten J.C., Koudstaal P.J., Visser M.C., Schouten H.J.A., van Gijn J. (1988). Interobserver agreement for the assessment of handicap in stroke patients. Stroke.

[25] Rinaldi P., Mecocci P., Benedetti C., Ercolani S., Bregnocchi M., Menculini G., Catani M., Senin U., Cherubini A. (2003). Validation of the five-item geriatric depression scale in elderly subjects in three different settings. J. Am. Geriatr. Soc..

[26] Folstein M.F., Folstein S.E., McHugh P.R. (1975). Mini-mental state. A pratical method for grading the cognitive state of patients for the clinician. J. Psychiatr. Res..

[27] Morris J.C. (1997). Clinical Dementia Rating: A Reliable and Valid Diagnostic and Staging Measure for Dementia of the Alzheimer Type. Int. Psychogeriatr..

[28] Jutai J., Day H. (2002). Psychosocial Impact of Assistive devices Scale (PIADS). Technol. Disabil..

[29] Mahoney F.I., Barthel D.W. (1965). Functional Evaluation: The Barthel Index. Md. State Med. J..

[30] Tinetti M.E. (1986). Performance-oriented assessment of mobility problems in elderly patients. J. Am. Geriatr. Soc..

[31] Ware J.E., Kosinski M., Keller S.D. (1998). SF-12: How to Score the SF-12 Physical and Mental Health Summary Scales.

[32] Ruggiero C., Mariani T., Gugliotta R., Gasperini B., Patacchini F., Nguyen H.N., Zampi E., Serra R., Dell’aquila G., Cirinei E. (2009). Validation of the Italian version of the falls efficacy scale international (FES-I) and the SHORT FES-I in community dwelling older persons. Arch. Gerontol. Geriatr..

[33] Low D.C., Walsh G.S. (2022). The minimal important change for measures of balance and postural control in older adults: A systematic review. Age Ageing.

[34] Jahantabi-Nejad S., Azad A. (2019). Predictive accuracy of performance oriented mobility assessment for falls in older adults: A systematic review. Med. J. Islam. Repub. Iran.

[35] Maranesi E., Di Donna V., Pelliccioni G., Cameriere V., Casoni E., Baldoni R., Benadduci M., Rinaldi N., Fantechi L., Giammarchi C. (2022). Acceptability and Preliminary Results of Technology-Assisted Balance Training in Parkinson’s Disease. Int. J. Environ. Res. Public Health.

[36] Bevilacqua R., Benadduci M., Riccardi G.R., Melone G., La Forgia A., MacChiarulo N., Rossetti L., Marzorati M., Rizzo G., Di Bitonto P. (2022). SI-ROBOTICS System: A preliminary study on usability of a rehabilitation program in patients with Parkinson’s disease. CEUR Workshop Proceedings.

[37] Cano-de-la-Cuerda R., Blázquez-Fernández A., Marcos-Antón S., Sánchez-Herrera-Baeza P., Fernández-González P., Collado-Vázquez S., Jiménez-Antona C., Laguarta-Val S. (2024). Economic Cost of Rehabilitation with Robotic and Virtual Reality Systems in People with Neurological Disorders: A Systematic Review. J. Clin. Med..

[38] Zhang B., Wong K.P., Kang R., Fu S., Qin J., Xiao Q. (2023). Efficacy of Robot-Assisted and Virtual Reality Interventions on Balance, Gait, and Daily Function in Patients With Stroke: A Systematic Review and Network Meta-analysis. Arch. Phys. Med. Rehabil..

[39] Lin Y., Li Q.Y., Qu Q., Ding L., Chen Z., Huang F., Hu S., Deng W., Guo F., Wang C. (2022). Comparative Effectiveness of Robot-Assisted Training Versus Enhanced Upper Extremity Therapy on Upper and Lower Extremity for Stroke Survivors: A Multicentre Randomized Controlled Trial. J. Rehabil. Med..

[40] Bang D.H., Shin W.S. (2017). Effects of an intensive Nordic walking intervention on the balance function and walking ability of individuals with Parkinson’s disease: A randomized controlled pilot trial. Aging Clin. Exp. Res..

[41] Bevilacqua R., Benadduci M., Bonfigli A.R., Riccardi G.R., Melone G., La Forgia A., Macchiarulo N., Rossetti L., Marzorati M., Rizzo G. (2021). Dancing With Parkinson’s Disease: The SI-ROBOTICS Study Protocol. Front. Public Health.

[42] Aras B., Seyyar G.K., Fidan O., Colak E. (2022). The effect of Tai Chi on functional mobility, balance and falls in Parkinson’s disease: A systematic review and meta-analysis of systematic reviews. Explore.

[43] Soke F., Guclu-Gunduz A., Kocer B., Fidan I., Keskinoglu P. (2021). Task-oriented circuit training combined with aerobic training improves motor performance and balance in people with Parkinson’s Disease. Acta Neurol. Belg..

[44] Picelli A., Melotti C., Origano F., Waldner A., Fiaschi A., Santilli V., Smania N. (2012). Robot-assisted gait training in patients with Parkinson disease: A randomized controlled trial. Neurorehabilit. Neural Repair.

[45] Fundarò C., Maestri R., Ferriero G., Chimento P., Taveggia G., Casale R. (2019). Self-selected speed gait training in Parkinson’s disease: Robot-assisted gait training with virtual reality versus gait training on the ground. Eur. J. Phys. Rehabil. Med..

[46] Mehdizadeh M., Martinez-Martin P., Habibi S.A., Fereshtehnejad S.M., Abasi A., Niazi Khatoon J., Saneii S.H., Taghizadeh G. (2019). Reliability and Validity of Fall Efficacy Scale-International in People with Parkinson’s Disease during On- and Off-Drug Phases. Park. Dis..

